# Oral SARS-CoV-2 Inoculation Causes Nasal Viral Infection Leading to Olfactory Bulb Infection: An Experimental Study

**DOI:** 10.3389/fcimb.2022.924725

**Published:** 2022-06-13

**Authors:** Rumi Ueha, Toshihiro Ito, Ryutaro Furukawa, Masahiro Kitabatake, Noriko Ouji-Sageshima, Satoshi Ueha, Misaki Koyama, Tsukasa Uranaka, Kenji Kondo, Tatsuya Yamasoba

**Affiliations:** ^1^ Swallowing Center, the University of Tokyo Hospital, Tokyo, Japan; ^2^ Department of Otolaryngology and Head and Neck Surgery, Faculty of Medicine, the University of Tokyo, Tokyo, Japan; ^3^ Department of Immunology, Nara Medical University, Nara, Japan; ^4^ Division of Molecular Regulation of Inflammatory and Immune Diseases, Research Institute for Biomedical Sciences, Tokyo University of Science, Chiba, Japan

**Keywords:** SARS-CoV-2, COVID-19, oral inoculation, olfactory dysfunction, olfactory epithelium, zone, olfactory bulb

## Abstract

Severe acute respiratory syndrome coronavirus 2 (SARS-CoV-2) infections can cause long-lasting anosmia, but the impact of SARS-CoV-2 infection, which can spread to the nasal cavity *via* the oral route, on the olfactory receptor neuron (ORN) lineage and olfactory bulb (OB) remains undetermined. Using Syrian hamsters, we explored whether oral SARS-CoV-2 inoculation can lead to nasal viral infection, examined how SARS-CoV-2 affects the ORN lineage by site, and investigated whether SARS-CoV-2 infection can spread to the OB and induce inflammation. On post-inoculation day 7, SARS-CoV-2 presence was confirmed in the lateral area (OCAM-positive) but not the nasal septum of NQO1-positive and OCAM-positive areas. The virus was observed partially infiltrating the olfactory epithelium, and ORN progenitor cells, immature ORNs, and mature ORNs were fewer than in controls. The virus was found in the olfactory nerve bundles to the OB, suggesting the nasal cavity as a route for SARS-CoV-2 brain infection. We demonstrated that transoral SARS-CoV-2 infection can spread from the nasal cavity to the central nervous system and the possibility of central olfactory dysfunction due to SARS-CoV-2 infection. The virus was localized at the infection site and could damage all ORN-lineage cells.

## Introduction

At the end of 2019, severe acute respiratory syndrome coronavirus 2 (SARS-CoV-2) was identified as the cause of a cluster of pneumonia cases in China, which rapidly spread, resulting in a global pandemic (Wang et al., 2020). SARS-CoV-2 infection (coronavirus disease; COVID-19) is associated with upper respiratory tract symptoms such as nasal discharge and sore throat, spreading to the lower respiratory tract, resulting in pneumonia, which in severe cases may require artificial respiration management (Jiang et al., 2020; Wang et al., 2020).

The characteristic symptoms of SARS-CoV-2 infection (COVID-19) comprise sensory impairments such as olfactory and gustatory dysfunctions. Among these, olfactory dysfunction is seen in 20–85% of patients (Costa et al., 2020; Han et al., 2020; Ibekwe et al., 2020), and more patients present with anosmia (complete loss of the sense of smell) than with partial olfactory disturbance (Butowt and von Bartheld, 2021; Saussez et al., 2021).

The coronavirus receptor proteins including angiotensin-converting enzyme 2 (ACE2) (Hoffmann et al., 2020) and neuropilin−1 (NRP1) (Cantuti-Castelvetri et al., 2020), as well as proteases such as transmembrane protease serine 2 (TMPRSS2) (Hoffmann et al., 2020) and Furin (Walls et al., 2020), which assist the entry of the virus into the tissues, are found in the upper respiratory tract, including the tongue, nasal cavity, and pharynx (Sato et al., 2021; Ueha et al., 2021). Therefore, the nasal and oral cavities are considered to be the major routes of SARS-CoV-2 infection. Although the mechanisms underlying olfactory dysfunction related to COVID-19 have been gradually elucidated through histological and genomic examinations (Singh et al., 2020; Butowt and von Bartheld, 2021; Sato et al., 2021; Ueha et al., 2021) and imaging studies (Naeini et al., 2020; Keshavarz et al., 2021), it remains difficult to disentangle the involved mechanisms because of the heterogeneity of presentations (Xydakis et al., 2021). Currently, the following mechanisms are being considered: 1) transient conductive olfactory dysfunction resulting from airway blockage due to local inflammation and edema of the nasal mucosa in the olfactory cleft and nasal passages; 2) temporary sensorineural olfactory dysfunction due to damage to supporting tissues such as the sustentacular cells, Bowman’s gland, and microvillar cells, excluding mature olfactory receptor cells (ORNs); 3) chronic or permanent sensorineural olfactory dysfunction due to damage to the ORNs; 4) central olfactory dysfunction due to SARS-CoV-2 infection of the olfactory bulb (OB) and olfactory cortex (Xydakis et al., 2021; Ye et al., 2021; Zhang et al., 2021).

Several studies using tissue samples from SARS-CoV-2-infected hamsters and humans have been reported to elucidate the mechanism of olfactory dysfunction (Bryche et al., 2020; de Melo et al., 2021; Seo et al., 2021; Urata et al., 2021; Zhang et al., 2021). Mouse species have been evaluated with SARS-CoV-2, but most do not show severe clinical signs because of the disparity between mouse and human ACE2 (Lee and Lowen, 2021). In contrast, the Syrian hamster ACE2 is highly homologous to human ACE2 and efficiently binds to the SARS-CoV-2 receptor binding domain; therefore, the Syrian hamster is recognized as a good small animal model for SARS-CoV-2 infection (Imai et al., 2020; Lee and Lowen, 2021). In Syrian hamsters, the olfactory epithelium (OE) is extensively disrupted in the first few days after SARS-CoV-2 infection, and the loss of microvilli becomes significant on days 2–7 (Seo et al., 2021; Urata et al., 2021). The virus mainly infects the sustentacular and microvillar cells and not the ORNs in the OE (Bryche et al., 2020; Seo et al., 2021). Subsequently, immune cells are recruited and infiltrate the OE and lamina propria of the olfactory mucosa (OM) (Bryche et al., 2020; de Melo et al., 2021). OE regeneration begins at approximately day 8 and is almost restored by day 21 in the nasal septum, while regeneration is insufficient in the lateral and dorsal regions (Urata et al., 2021). This means that the regeneration process varies depending on the location in the nasal cavity. The ORNs are classified into four groups according to their zonal expression patterns, and odorant receptors are expressed by sensory neurons distributed within one of four circumscribed zones (Yoshihara et al., 1997; Mori et al., 2000; Imamura and Hasegawa-Ishii, 2016). Of those, zone 1 is determined by co-localization with NQO1 expression and zones 2–4 are determined by OCAM expression (Yoshihara et al., 1997; Mori et al., 2000; Gussing and Bohm, 2004; Imamura and Hasegawa-Ishii, 2016). However, the impacts of various viral infections on the ORNs, including ORN progenitor cells and immature ORNs, have not been well established, and especially, the effects of SARS-CoV-2 infection on each zone of the OE have not been verified. Additionally, the effects of SARS-CoV-2 infection on the OB, the center of olfaction, are poorly understood, although they can cause long-lasting and complete loss of smell (Butowt and von Bartheld, 2021; Saussez et al., 2021). It is imperative to uncover the routes of SARS-CoV-2 infection and transmission to prevent and develop treatments for this sensory disorder that reduces the quality of life.

Although it is unlikely that the nasal route is actually the only SARS-CoV-2 nasal infection route, the transnasal route of SARS-CoV-2 is often applied in experimental models to investigate olfactory disorders. It is conceivable that SARS-CoV-2 may also spread to the nasal cavity *via* the oral route, but this possibility has not been well verified. Therefore, clarification is needed to determine whether oral infection can also transmit the virus to the OM and OB.

In this study, we first explored whether oral SARS-CoV-2 inoculation, not nasal administration, can lead to nasal viral infection, especially to the olfactory related tissue, using hamster models. Secondly, we examined how SARS-CoV-2 affects the OE, including ORN-related cells, by site. Third, we also investigated whether SARS-CoV-2 infection can spill over to the OB and induce inflammation.

## Methods

### Animals

Six-week-old, male Syrian Golden hamsters (*Mesocricetus auratus*) were purchased from Japan SLC (Hamamatsu, Shizuoka, Japan) and maintained in a specific pathogen-free environment at the Animal Research Center of Nara Medical University. The animal experimental protocols were in compliance with the ARRIVE guidelines, and were approved by The Animal Care and Use Committee at Nara Medical University (approval number, 12922). All procedures were performed in compliance with relevant guidelines on the Care and Use of Laboratory Animals, Nara Medical University and Animal Research and the Animal Care and Use Committee of the University of Tokyo.

### Infection Model Preparation

The SARS-CoV-2 strain (JPN/TY/WK-521) was isolated and provided by the National Institute of Infectious Diseases, Japan. Virus culture was performed using VeroE6/TMPRSS2 cells (JCRB Cell Bank in Japan, JCRB1819). Hamsters were distributed into two groups. Before virus inoculation, they were anesthetized with an intraperitoneal injection of pentobarbital (10 mg/mL, 0.8mL/hamster). In the infection group, 50 μL of virus solution diluted with saline (including 1.0×10^5^ pfu of SARS-CoV-2) was administered into the mouth following the method of the previous paper (Furukawa et al., 2021). In the control group, 50 μL of saline was administered. Considering that weight loss and lung tissue damage in the Syrian hamster models were stronger on days 6-8 post-infection, the evaluation time point for the noses and OBs was set at day 7 post-infection in this study (Imai et al., 2020). At 7 days after inoculation, the SARS-CoV-2-challenged hamsters were euthanized by intraperitoneal injection of 1.0 ml sodium pentobarbital (10 mg/mL) followed by cardiac exsanguination. The noses and OBs were sampled for histopathological examination and the lungs were sampled for histopathological examination and quantitative polymerase chain reaction (qPCR). All experiments using SARS-CoV-2 were performed at the biosafety level 3 experimental facility of Nara Medical University, Japan.

### RNA Extractions and RT-qPCR

We verified SARS-CoV-2 viral RNA in the lungs to confirm that each hamster was infected with SARS-CoV-2. Total RNA was isolated from the lung using NucleoSpin^®^ RNA (Macherey-Nagel, Düren, Germany), then converted to cDNA using a High-Capacity cDNA Reverse Transcription Kit (Thermo Fisher Scientific, Waltham, MA, USA), according to the manufacturer’s instructions. RT-qPCR analysis was performed using a StepOnePlus Real-Time PCR System (Thermo Fisher Scientific). The gene-specific primers and probes used were: *Gapdh* as endogenous control (TaqMan assay Cg04424038) and SARS-CoV-2 nucleocapsid gene (forward: 5’- AAATTTTGGGGACCAGGAAC -3’, reverse: 5’- TGGCAGCTGTGTAGGTCAAC -3’, the TaqMan probe: FAM-ATGTCGCGCATTGGCATGGA-BHQ). The expression levels of each gene were normalized to the level of *Gapdh* expression for each sample.

### Tissue Preparation

Immediately after tissue harvesting, the lungs and nasal tissues were gently irrigated and fixed in 4% paraformaldehyde for 14 days. Then, the tissue samples were decalcified, dehydrated in graded ethanol solutions, and embedded in paraffin. For histological analysis of the OM, coronal sections were obtained from all samples at the level of the anterior end of the OB (Ueha et al., 2016; Ueha et al., 2016; Ueha et al., 2018; Ueha et al., 2018). Four-micrometer-thick paraffin sections were deparaffinized in xylene and rehydrated in ethanol before staining.

### Histological Analyses

Hematoxylin and eosin staining was performed to evaluate the overall tissue structure. For immunostaining, deparaffinized sections were treated with 3% hydrogen peroxide to block endogenous peroxidase activity, and then incubated in Blocking One solution (Nacalai Tesque, Kyoto, Japan) to block non-specific immunoglobulin binding. After antigen retrieval, the samples were incubated with primary antibodies, followed by secondary antibodies.

The primary and secondary antibodies used in this study are listed in **Table 1**. Anti-NQO1 and anti-OCAM antibodies were used to confirm the location of zone 1 or zones 2–4. Anti-SARS-CoV-2 nucleocapsid antibody was used to identify SARS-CoV-2. The following antibodies were used to evaluate ORN neurogenesis: sex-determining region Y-box 2 (SOX2), expressed by proliferating stem cells or progenitor cells in the basal layer of the OE; growth-associated protein 43 (GAP43), expressed by immature ORNs in the OE; olfactory marker protein (OMP), expressed by mature ORNs in the OE. Ki67 and cleaved caspase-3 (Cas3) were used as cellular markers for proliferation and cell death, separately. To assess inflammatory cell infiltration, CD3 and MPO were used for detection of T cells and for polymorphonuclear neutrophils and macrophages, respectively (Loria et al., 2008; Ueha et al., 2019). Ionized calcium-binding adaptor molecule 1 (Iba1), a microglial marker, was applied as an indicator of the inflammatory response in the OB (Lazarini et al., 2012).

**Table 1:** The primary and secondary antibodies used in this study.

Primary antibody	Source	Catalog No.	Host	Type	Dilution
OCAM	R&D Systems (Minneapolis, MN, USA)	AF778	Goat	polyclonal	1:200
NQO1	Abcam (Cambridge, UK),	ab34173	Rabbit	polyclonal	1:500
SARS-CoV-2 nucleocapsid	GeneTex(Irvine, CA, USA)	GTX135357	Rabbit	polyclonal	1:1000
SOX2	Abcam (Cambridge, UK),	ab92494	Rabbit	monoclonal	1:300
GAP43	Novus Biologicals (Centennial, CO, USA)	NB300-143B	Rabbit	polyclonal	1:1000
OMP	Wako (Tokyo, Japan)	019-22291	Goat	polyclonal	1:8000
CD3	Nichirei (Tokyo, Japan)	413601	Rabbit	monoclonal	1:300
MPO	Abcam (Cambridge, UK),	ab9535	Rabbit	polyclonal	1:150
PGP9.5	Dako (Carpinteria, CA, USA)	Z5116	Rabbit	polyclonal	1:1000
Ki67	Novus Biologicals (Centennial, CO, USA)	NB600-1252	Rabbit	monoclonal	1:200
Caspase-3	Cell Signaling (Tokyo, Japan)	9661	Rabbit	polyclonal	1:300
IBA1	Proteintech (Tokyo, Japan)	10904-1-AP	Rabbit	polyclonal	1:1000
Secondary antibody					
Anti-rabbit IgG	Thermo Fisher scientific (Tokyo, Japan)	A21207	Donkey	polyclonal	1:1000
Anti-goat IgG	Thermo Fisher scientific (Tokyo, Japan)	A11055	Donkey	polyclonal	1:1000
Anti-rabbit IgG (HRP)	Thermo Fisher scientific (Tokyo, Japan)	31460	Goat	polyclonal	1:5000
Anti-goat IgG (HRP)	Thermo Fisher scientific (Tokyo, Japan)	31402	Rabbit	polyclonal	1:3000

**Figure 1:**
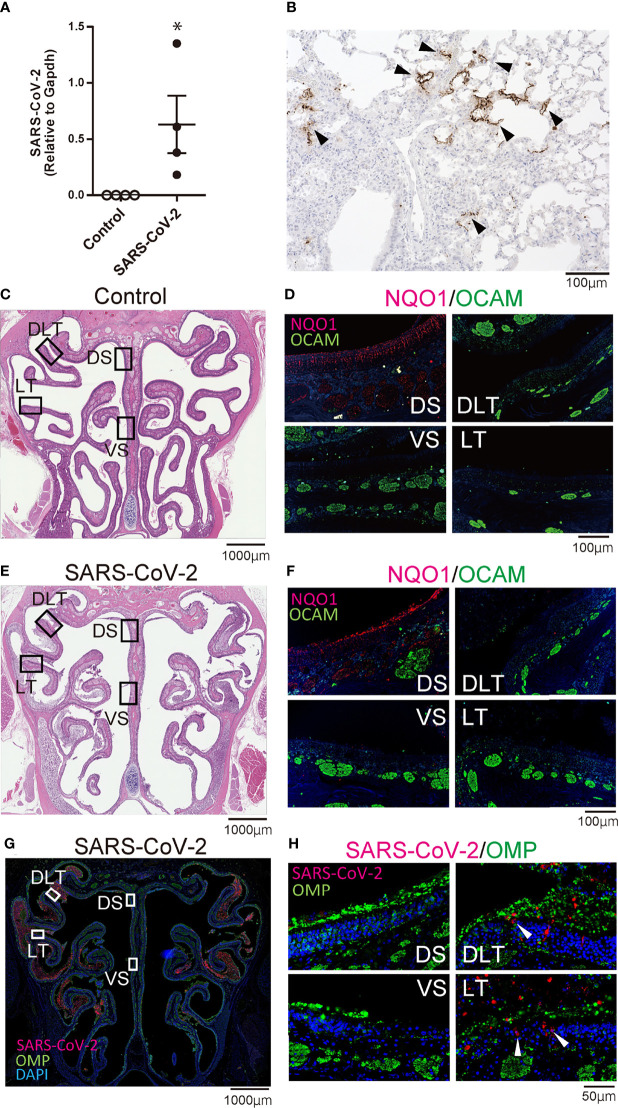
Infectious findings in the lungs and nasal cavity. **(A)** Severe acute respiratory syndrome coronavirus 2 (SARS-CoV-2) gene detection with RT- quantitative polymerase chain reaction (*P < 0.05). **(B)** Immunohistochemistry staining of SARS-CoV-2 in the lung (shown in brown, arrow heads). **(C, D)** Representative images of the olfactory epithelium from control hamsters (C: hematoxylin and eosin stain, D: immunohistological staining of NQO1 and OCAM). The boxes in **(C)** indicate the regions of the olfactory epithelium shown in D; the dorsal nasal septum (DS) area, ventral nasal septum (VS) area, dorsal lateral turbinate (DLT) area, and lateral turbinate (LT) area. Only the DS area appears positive for NQO1. **(E, F)** Representative images of the olfactory epithelium from SARS-CoV-2 hamsters. **(G)** Immunohistochemistry staining of the olfactory epithelium in SARS-CoV-2 hamsters. The virus is mainly present in the lateral region of the nasal cavity. **(H)** Double stained images of SARS-CoV-2 and OMP in each region of the olfactory epithelium. No double positive cells for SARS-CoV-2 and OMP are present in the olfactory epithelium. The arrow heads show SARS-CoV-2 positive cells.

To analyze the OE, coronal sections of the OE were divided into four areas along the zonal organization: the dorsal nasal septum (DS) area, ventral nasal septum (VS) area, dorsal lateral turbinate (DLT) area, and lateral turbinate (LT) area (**Figure 1A**). To analyze the OB, coronal sections of the OB-surrounding tissue were divided into three areas: the cribriform plate area, medial area, and lateral area.

Images were captured using a digital microscope camera (Keyence BZ-X700, Osaka, Japan) with 4×, 20×, and 40× objective lenses. OMP^+^ ORNs, SOX2^+^ ORN progenitors, GAP43^+^ immature ORNs, and Ki67^+^ cells in a 300-μm region of each area were counted in the right and left sides of each sample. The number of each cell type was quantitatively analyzed using sections with single or double immunostaining for each antigen and counterstaining with hematoxylin or DAPI.

### Statistical Analysis

Statistical comparisons between groups were performed with the Kolmogorov-Smirnov test or the Mann–Whitney U-test using GraphPad Prism software (version 6.7; GraphPad Software, Inc., San Diego, CA, USA, www.graphpad.com). qPCR data were subjected to logarithmic transformation prior to analysis. Results with *P* < 0.05 were considered statistically significant.

## Results

### Oral SARS-CoV-2 Inoculation Induces Nasal Infection in a Zone/Site-Dependent Manner

To confirm that SARS-CoV-2 infection was established, we first evaluated the presence of the virus in the lungs with RT-qPCR and immunohistochemistry. The virus was identified histologically and with RT-qPCR in the lungs of SARS-CoV-2-infected hamsters on day 7 after oral inoculation, and SARS-CoV-2 infection was observed in all hamsters orally inoculated (**Figures 1A, B**). Next, we investigated the distribution of each zone in the OM based on the expression patterns of specific molecules including NQO1 and OCAM. In the OM of control hamsters, the DS area was NQO1 positive (zone 1) and all other areas (VS, DLT, LT) were OCAM positive (zone 2-4) (**Figures 1C, D**). Similarly, in SARS-CoV-2 hamsters, the OE could be divided into NQO1-positive and OCAM-positive regions (**Figures 1E, F**). Then, we examined the nasal cavity for the presence of SARS-CoV-2 through immunostaining on day 7 after oral virus inoculation. Although SARS-CoV-2 was not detected in the NS (NQO1-positive) and VS (OCAM-positive) areas of the nasal cavity of infected hamsters, the virus was extensively confirmed in the DLT and LT (OCAM-positive) areas. The virus was observed infiltrating a part of the OE and predominantly around OMP-negative cells in the OE (**Figures 1G, H**; **Supplementary Figure 1**).

### SARS-CoV-2 Infection Reduces the Olfactory Receptor Neurons at Various Differentiation Stages

**Figure 2:**
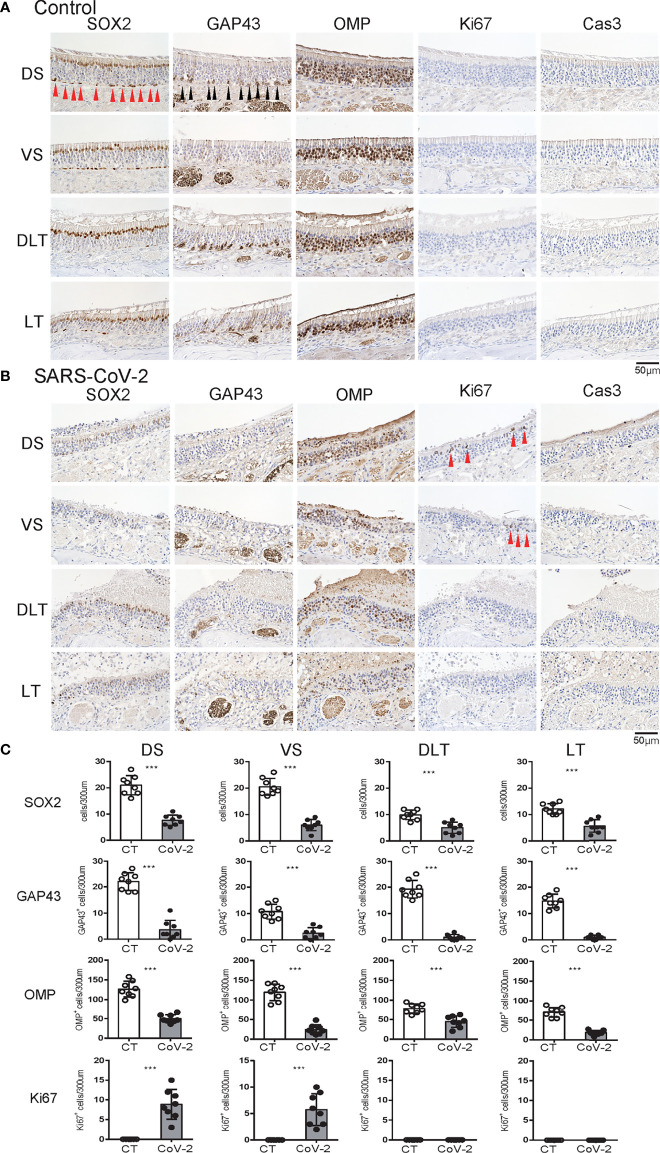
Effects of SARS-CoV-2 infection on the olfactory receptor neuron lineage. **(A, B)** Representative images of immunohistological staining in control hamsters **(A)** and severe acute respiratory syndrome coronavirus 2 (SARS-CoV-2) hamsters **(B)**. Sex-determining region Y-box 2 (SOX2)^+^ progenitor cells, growth-associated protein 4 (GAP43)^+^ immature olfactory receptor neurons (ORNs), olfactory marker protein (OMP)^+^ ORNs, Ki67^+^ proliferating cells, and cleaved caspase-3 (Cas3)^+^ apoptotic cells are shown in brown. Each cell, except for the numerous OMP+ cells, is indicated by arrows. Tissue sections were counterstained with the nuclear dye hematoxylin (blue). **(C)** Numbers of SOX2^+^ ORN progenitors and Ki67^+^ actively proliferating cells per 300 μm of the basal layer and OMP+ mature ORNs, GAP43+ immature ORNs, and Cas3+ apoptotic cells per 300 μm of olfactory epithelium in each area are counted in control or SARS-CoV-2 hamsters. ***P < 0.001.

**Figure 3:**
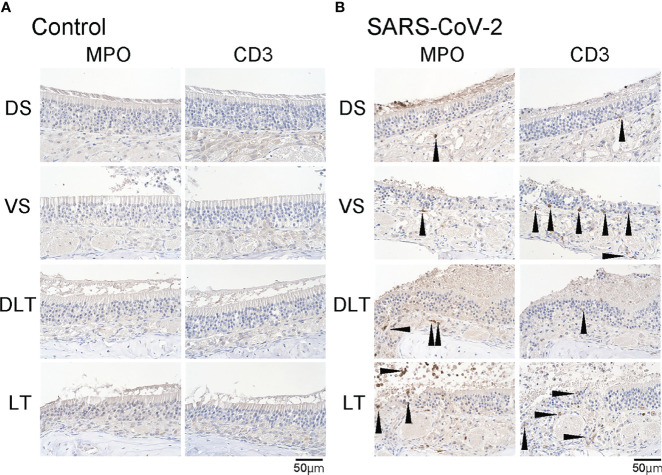
Inflammatory cell infiltration in the olfactory mucosa. **(A, B)** Representative images of immunohistological staining of MPO and CD3 in control hamsters **(A)** and severe acute respiratory syndrome coronavirus 2 (SARS-CoV-2) hamsters **(B)**. **(B)** In the SARS-CoV-2 group, some MPO^+^ and CD3^+^ cells are present in the olfactory epithelium.

We subsequently examined the effect of SARS-CoV-2 infection on the ORN lineage. The numbers of SOX2^+^ ORN progenitor cells, GAP43^+^ immature ORNs, and OMP^+^ mature ORNs were lower in the infected group than in the control group on day 7 after SARS-CoV-2 infection in all four areas. In the control group, the middle layer of the OE was almost completely filled with OMP^+^ ORNs, whereas in the SARS-CoV-2 infected group, OMP-negative cells were scattered in the middle layer of the OE to varying degrees, and these cells were negative for both GAP43 and SOX2 (**Figure 2**). Ki67^+^ dividing cells were found in the most superficial layer of the OE in the DS and VS areas, but not in the strongly infected lateral regions (DLT and LT). Few apoptotic cells were found in the entire nasal cavity. Regarding inflammatory cell infiltration, some MPO^+^ cells were found in the DLT and LT regions, where SARS-CoV-2 was found, but a few CD3 positive cells were present in the VS, LT, and MT regions. In brief, the DS region showed the least inflammatory cell infiltration on day 7 after SARS-CoV-2 infection (**Figure 3**).

### SARS-CoV-2 Can Invade the Olfactory Bulb From the Olfactory Epithelium *Via* the Olfactory Nerve

**Figure 4:**
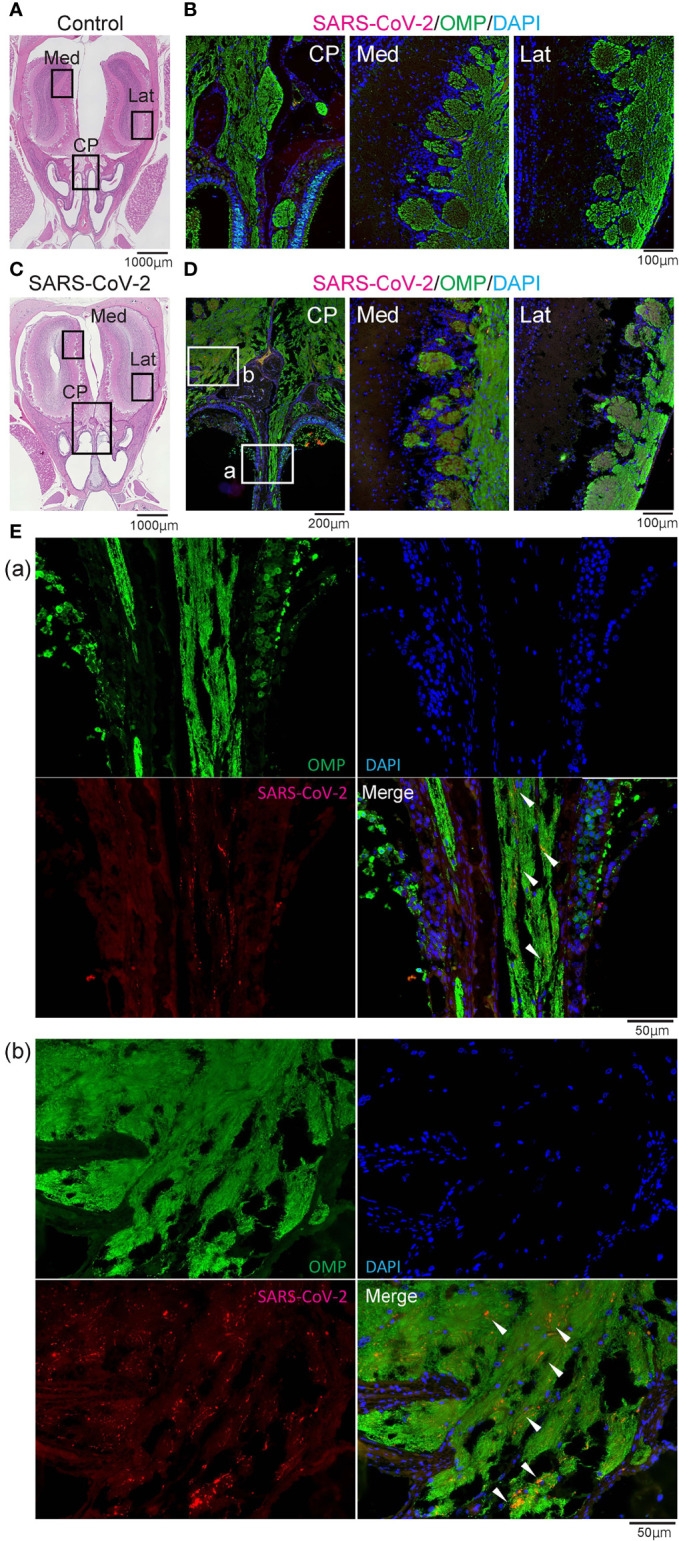
Representative images of SARS-CoV-2 and OMP staining in the olfactory nerve bundles and olfactory bulb. Representative coronal section images of the olfactory bulb area. **(A, B)** Images of control hamsters (A: hematoxylin and eosin stain, B: immunohistological staining of severe acute respiratory syndrome coronavirus 2 (SARS-CoV-2), olfactory marker protein (OMP), and DAPI). The boxes in **(A)** indicate the areas shown enlarged in B; the cribriform plate area (CP), medial area (Med), and lateral area (Lat). **(C–E)** Images of SARS-CoV-2 hamsters (**C**: hematoxylin and eosin stain, **D, E**: immunohistological staining of SARS-CoV-2, OMP, and DAPI). The boxes in **(C)** indicate the areas shown enlarged in **(D)**, and the boxes in **(D)** are shown enlarged in **(E)**. SARS-CoV-2 is present in the olfactory nerve of the nasal mucosa and the olfactory nerve fiber layer around the olfactory bulb.

**Figure 5:**
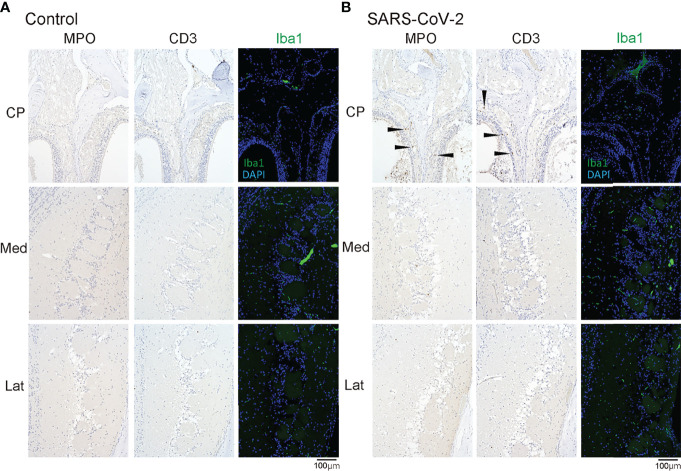
MPO/CD3/Iba1 positive cells in the olfactory bulb area. Representative immunohistological images of MPO and CD3 in the olfactory bulb area (**A**: control hamster, **B**: SARS-CoV-2 hamster). Some MPO^+^ cells and CD3^+^ cells are observed in the olfactory mucosa around the cribriform plate (arrow heads). CP, cribriform plate area; Iba1, ionized calcium-binding adaptor molecule 1; Lat, lateral area; Med, medial area; SARS-CoV-2, severe acute respiratory syndrome coronavirus 2.

Last, we examined whether SARS-CoV-2 could enter the central nervous system through the nasal cavity. The olfactory nerve fibers, which transmit odor information from the ORNs, penetrate small holes in the cribriform plate of the ethmoid bone and reach the OB. In the SARS-CoV-2 infected group, the virus was found in the olfactory nerve on the nasal side and the olfactory nerve fiber layer on the OB side, suggesting that the nasal cavity is a route to brain infection in COVID-19. In addition, a small amount of SARS-CoV-2 was seen in the medial region of the OB (**Figure 4**). However, there was minor inflammatory cell infiltration in the OB, and only few MPO^+^ and CD3^+^ cells were observed in the lamina propria of the OM close to the cribriform plate. No obvious increase in Iba1^+^ activated microglia was observed in the OB of the SARS-CoV-2 group (**Figure 5**).

## Discussion

The present study showed that SARS-CoV-2 infection *via* the oral route can spread to the nasal cavity and OB in Syrian hamsters. SARS-CoV-2 was detected in the nasal cavity 7 days after oral SARS-CoV-2 inoculation, but the virus infection site was mainly on the lateral side of the nasal cavity. The numbers of ORN-associated cells were reduced in all lineages. SARS-CoV-2 was identified in the olfactory nerve bundles in the pathway from the OM to the OB.

Although the infection conditions in the nasal cavity may be different between the transnasal and transoral infection models of SARS-CoV-2, the virus oral inoculation model may show an infection situation more similar to the actual living environment than the virus nasal administration model. The findings that SARS-CoV-2 entering the host animals *via* the oral route not only infected the lower respiratory tract, but also the nasal tissues and induced degeneration of the OE, is an important message regarding infection countermeasures in daily life. Although SARS-CoV-2 has been previously identified in the nasal turbinate of orally virus-infected hamsters (Lee et al., 2020), infection of the olfactory-related tissue has not been investigated. The present study revealed that even oral infection can spread to olfactory-related tissues including the OE and OB. By inhaling droplets from conversations during eating and drinking, or by eating something touched with SARS-CoV-2-contaminated hands, the virus is likely to adhere to the oral cavity and enter the body, resulting in olfactory dysfunction in some people. Therefore, individuals should thoroughly wash their hands before eating, be careful of splashing droplets during conversation while eating, and avoid sharing meals with others to prevent infection.

Regarding the localization of SARS-CoV-2 infection sites in the nasal cavity and differences in the OE damage depending on the site, a previous report showed that it is more likely for SARS-CoV-2 to be found in the lateral region where there is a greater degree of tissue damage (Urata et al., 2021), which is consistent with the present results. In addition, this is also analogous to the fact that the OE in the dorsolateral region is susceptible to damage *via* allergic inflammation (Ueha et al., 2020). Conversely, it has been reported that the NQO1-positive OE is more likely to be damaged by long-term exercise and caloric restriction (Tuerdi et al., 2018). Thus, it is suggested that the susceptibility of the OM to damage depends on the type of damage and the location of the OE.

Many studies have examined which OE cells are susceptible to infection by SARS-CoV-2, and it is now considered that the virus primarily infects the olfactory cilia and supporting cells but can also infect some basal ORN progenitors (Bryche et al., 2020; Cantuti-Castelvetri et al., 2020; de Melo et al., 2021; Seo et al., 2021). However, SARS-CoV-2 can also affect olfactory neurons, albeit slightly (de Melo et al., 2021). ACE2, a receptor for SARS-CoV-2, is mainly expressed in the supporting cells and basal cells of the OE, while the mature ORNs rarely express ACE2 (Brann et al., 2020; Ueha et al., 2021). However, considering that NRP1, the other SARS-CoV-2 receptor is found in almost all cell types of the OE (Cantuti-Castelvetri et al., 2020; Ziuzia-Januszewska and Januszewski, 2022), it is unsurprising that almost the entire OE is impaired by SARS-CoV-2 infection. In the present validation, the numbers of all ORN-related cells, including ORN progenitors, immature ORNs, and mature ORNs, were reduced in each area of the OE compared to the control group, although differences were observed in each area of the OE. Although the present study was limited to the analysis of day 7 after oral SARS-CoV-2 inoculation, and the temporal changes before and after the infection could not be confirmed, we discuss the following.

In the DS and VS areas where the virus could not be identified, the epithelial layer of the SARS-CoV-2 group was thinner than that of the control group. The numbers of basal cells, immature ORNs, and mature ORNs were reduced, and only the cells in the superficial layer of the OE were dividing, suggesting that the OE was damaged after virus infection and that it may have been in the regeneration stage at the time of examination. As GAP43^+^ immature ORNs could not be identified and Ki67^+^ cells were observed in the superficial layer, rather than in the basal layer, we speculate that the time point we verified in this study was the stage when the damaged sustentacular cells had first started to regenerate and that the basal cells (SOX2^+^ ORN progenitors) and their differentiation process to GAP43^+^ immature ORNs are suppressed by SARS-CoV-2 infection. Considering that inflammatory cell infiltration is not as abundant in the OM, it is presumed that the OE damage was induced by direct cell damage caused by the virus rather than by the effects of inflammatory cell infiltration. In the previously reported transnasal infection model of SARS-CoV-2, SARS-CoV-2 mainly infected the sustentacular cells, not the ORNs, and most of their cilia were lost early after infection (Bryche et al., 2020; Seo et al., 2021). It was also reported that SARS-CoV-2 could only be identified in the nasal septum of intranasal infected hamsters up to 3 days after infection (Urata et al., 2021), which is consistent with our results. In the nasal septum of this animal model, a large proportion of the OE was damaged within a few days after infection, followed by epithelial regeneration over time (Urata et al., 2021). Although the temporal changes after infection in the oral SARS-CoV-2 inoculation model may differ from those in the transnasal infection model, we speculate that the DS and VS areas, where no SARS-CoV-2 was identified in this study, will regenerate their epithelial tissue.

In the DLT and LT areas where the virus was present, while sustentacular cells and basal ORN progenitors could be seen to some extent, immature ORNs could hardly be seen, and mature ORNs could only be sparsely seen. As several cells negative for SOX2, GAP43, and OMP were found in the middle layer of the OE, it is possible that SARS-CoV-2 impaired the function of mature ORNs and prevented normal ORN protein expression. Taken together, the possible effects of SARS-CoV-2 infection on the ORNs are as follows: (1) sustentacular cells cannot support the ORNs because of viral infection, resulting in difficulty in maintaining the epithelial morphology, reduced function of mature ORNs, and disturbances in odor perception (Brann et al., 2020; Bryche et al., 2020; Cantuti-Castelvetri et al., 2020; de Melo et al., 2021; Seo et al., 2021); (2) SARS-CoV-2 directly infects the ONRs and reduces their function, although the percentage may be small (de Melo et al., 2021); and (3) SARS-CoV-2 may inhibit the maturation and differentiation of immature ORNs into mature ORNs.

Although the olfactory route is considered a potential means for SARS-CoV-2 invasion into the central nervous system (Jiao et al., 2021), there are other several potential pathways for the invasion of SARS-COV-2 to the central nervous system, such as the lymphatic and hematogenous route, and the gut-brain axis communication route (Kumar et al., 2020; Xu et al., 2021). In this study, we found SARS-CoV-2 in the olfactory nerve bundle around the cribriform plate, indicating that the nasal cavity is an entry route for the virus into the central nervous system. The other study using Syrian golden hamsters reported prolonged and extended impacts of SARS-CoV-2 on the olfactory neurocircuit such as prolonged activation of glial cells in the OB, and a decrease in the density of dendritic spines within the hippocampus (Kishimoto-Urata et al., 2022). In human studies, SARS-CoV-2 has been detected in the OB of autopsied cases (Morbini et al., 2020; Meinhardt et al., 2021). Imaging studies have also shown hyperintensity of the OB on T2 fluid-attenuated inversion recovery magnetic resonance images (Strauss et al., 2020; Keshavarz et al., 2021) and a pattern of hypometabolism involving the olfactory system (orbitofrontal cortex and olfactory and rectus gyri) on positron emission tomography of brain (Najt et al., 2021). A study on rhesus monkeys reported that SARS-CoV-2 primarily invades the central nervous system *via* the OB (Jiao et al., 2021). In human angiotensin-converting enzyme 2 transgenic mice infected with SARS-CoV-2, viral RNA was detected throughout the OB, including in the olfactory nerve layer, 7 days after infection (Golden et al., 2020). Approximately 10% of patients continued to experience olfactory dysfunction more than 6 months after SARS-CoV-2 infection (Boscolo-Rizzo et al., 2021; Kapoor et al., 2021), suggesting that the virus affected the central olfactory system, and the results of this study support the possibility of central olfactory dysfunction due to SARS-CoV-2 infection.

In conclusion, this study suggests that transoral SARS-CoV-2 infection may spread to the nasal cavity and then to the central nervous system through the olfactory route. To prevent SARS-CoV-2 infection in daily life, individuals should take adequate infection prevention measures and avoid performing activities associated with transoral infection.

## Data Availability Statement

The raw data supporting the conclusions of this article will be made available by the authors, without undue reservation.

## Ethics Statement

The animal study was reviewed and approved by The Animal Care and Use Committee at Nara Medical University (approval number, 12922).

## Author Contributions

RU developed the concept, designed and performed the experiments, analyzed the data, produced the figures, and wrote the initial draft of the manuscript. TI, RF, MK (4th Author), and NO-S prepared the animal models, performed some of the experiments, and analyzed the data. MK (7th Author) and TU performed some of the experiments and analyzed the data. KK and TY developed the concept and designed and critically revised the manuscript. All authors contributed to interpretation of the data and writing of the manuscript.

## Funding

This work was supported by JSPS KAKENHI Grant-in-Aid for Scientific Research (C) [grant number 19K09841], and MSD Life Science Foundation.

## Conflict of Interest

The authors declare that the research was conducted in the absence of any commercial or financial relationships that could be construed as a potential conflict of interest.

## Publisher’s Note

All claims expressed in this article are solely those of the authors and do not necessarily represent those of their affiliated organizations, or those of the publisher, the editors and the reviewers. Any product that may be evaluated in this article, or claim that may be made by its manufacturer, is not guaranteed or endorsed by the publisher.

## Supplementary Material

Supplementary Figure 1 Double stained images of SARS-CoV-2 and OMP in the olfactory epithelium of the control hamster. Double stained images of SARS-CoV-2 and OMP in each region of the olfactory epithelium. The boxes in **(A)** indicate the regions of the olfactory epithelium shown in **(B)**; the dorsal nasal septum (DS) area, ventral nasal septum (VS) area, dorsal lateral turbinate (DLT) area, and lateral turbinate (LT) area. No positive cells for SARS-CoV-2 are present in the olfactory epithelium.
