# Supplementary figures and images for: Oral SARS-CoV-2 Inoculation Causes Nasal Viral Infection Leading to Olfactory Bulb Infection: An Experimental Study
Source: Front Cell Infect Microbiol. 2022 Jun 13;12:924725. doi: 10.3389/fcimb.2022.924725 (PMC9234459; doi:10.3389/fcimb.2022.924725)

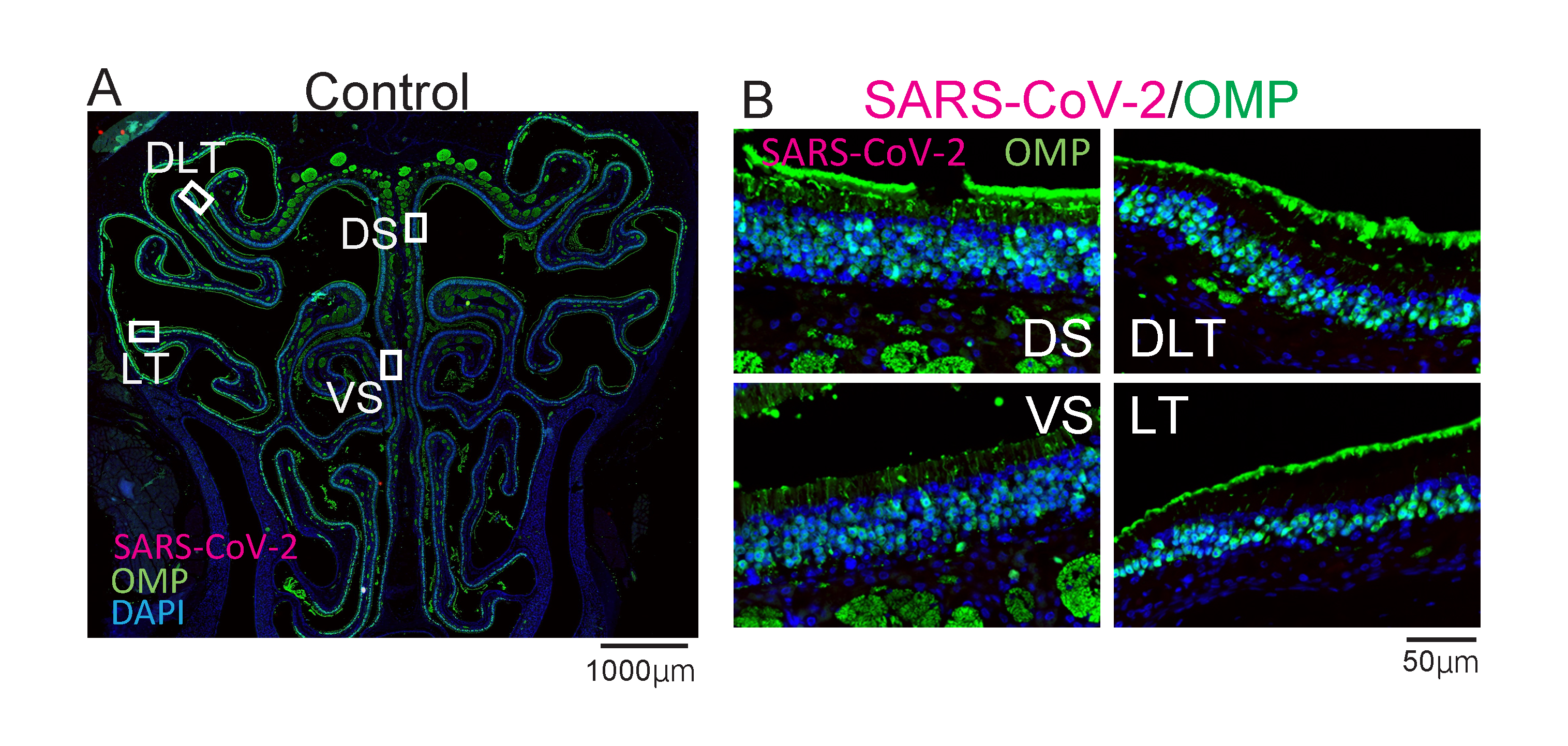

Supplement: Supplementary Figure 1 — Double stained images of SARS-CoV-2 and OMP in the olfactory epithelium of the control hamster. Double stained images of SARS-CoV-2 and OMP in each region of the olfactory epithelium. The boxes in (A) indicate the regions of the olfactory epithelium shown in (B); the dorsal nasal septum (DS) area, ventral nasal septum (VS) area, dorsal lateral turbinate (DLT) area, and lateral turbinate (LT) area. No positive cells for SARS-CoV-2 are present in the olfactory epithelium. [file Image_1.tiff]
